# A Physician-Completed Digital Tool for Evaluating Disease Progression (Multiple Sclerosis Progression Discussion Tool): Validation Study

**DOI:** 10.2196/16932

**Published:** 2020-02-12

**Authors:** Tjalf Ziemssen, Daniela Piani-Meier, Bryan Bennett, Chloe Johnson, Katie Tinsley, Andrew Trigg, Thomas Hach, Frank Dahlke, Davorka Tomic, Chloe Tolley, Mark S Freedman

**Affiliations:** 1 Center of Clinical Neuroscience Neurological University Clinic Carl Gustav Carus TU Dresden Dresden Germany; 2 Novartis Pharma AG Basel Switzerland; 3 Adelphi Values Macclesfield United Kingdom; 4 Ottawa Health Research Institute University of Ottawa Ottawa, ON Canada

**Keywords:** multiple sclerosis, relapsing-remitting multiple sclerosis, secondary progressive multiple sclerosis, transition, progression, digital, validation

## Abstract

**Background:**

Defining the transition from relapsing-remitting multiple sclerosis (RRMS) to secondary progressive multiple sclerosis (SPMS) can be challenging and delayed. A digital tool (MSProDiscuss) was developed to facilitate physician-patient discussion in evaluating early, subtle signs of multiple sclerosis (MS) disease progression representing this transition.

**Objective:**

This study aimed to determine cut-off values and corresponding sensitivity and specificity for predefined scoring algorithms, with or without including Expanded Disability Status Scale (EDSS) scores, to differentiate between RRMS and SPMS patients and to evaluate psychometric properties.

**Methods:**

Experienced neurologists completed the tool for patients with confirmed RRMS or SPMS and those suspected to be transitioning to SPMS. In addition to age and EDSS score, each patient’s current disease status (disease activity, symptoms, and its impacts on daily life) was collected while completing the draft tool. Receiver operating characteristic (ROC) curves determined optimal cut-off values (sensitivity and specificity) for the classification of RRMS and SPMS.

**Results:**

Twenty neurologists completed the draft tool for 198 patients. Mean scores for patients with RRMS (n=89), transitioning to SPMS (n=47), and SPMS (n=62) were 38.1 (SD 12.5), 55.2 (SD 11.1), and 69.6 (SD 12.0), respectively (*P*<.001, each between-groups comparison). Area under the ROC curve (AUC) including and excluding EDSS were for RRMS (including) AUC 0.91, 95% CI 0.87-0.95, RRMS (excluding) AUC 0.88, 95% CI 0.84-0.93, SPMS (including) AUC 0.91, 95% CI 0.86-0.95, and SPMS (excluding) AUC 0.86, 95% CI 0.81-0.91. In the algorithm with EDSS, the optimal cut-off values were ≤51.6 for RRMS patients (sensitivity=0.83; specificity=0.82) and ≥58.9 for SPMS patients (sensitivity=0.82; specificity=0.84). The optimal cut-offs without EDSS were ≤46.3 and ≥57.8 and resulted in similar high sensitivity and specificity (0.76-0.86). The draft tool showed excellent interrater reliability (intraclass correlation coefficient=.95).

**Conclusions:**

The MSProDiscuss tool differentiated RRMS patients from SPMS patients with high sensitivity and specificity. In clinical practice, it may be a useful tool to evaluate early, subtle signs of MS disease progression indicating the evolution of RRMS to SPMS. MSProDiscuss will help assess the current level of progression in an individual patient and facilitate a more informed physician-patient discussion.

## Introduction

Multiple sclerosis (MS) is the most common acquired chronic degenerative disease of the central nervous system in young adults, with more than 2.3 million people affected by the disease worldwide [1]. MS evolves as a continuum with an active initial relapsing-remitting course in most patients that, generally, gradually transitions to a phase of progressive accumulation of disability with or without continued activity—relapses or new inflammatory lesions [2,3]. Approximately 50% of patients with an initial relapsing-remitting course transition to the secondary progressive phase over 15 to 20 years [4,5]. The diagnosis of secondary progressive MS (SPMS) is challenging owing to a lack of accepted clinical, imaging, immunologic, or pathologic criteria to determine when relapsing-remitting MS (RRMS) converts to SPMS [6-8]. The diagnosis of SPMS is done retrospectively based on a history of gradual relapse-free progression over at least 6 to 12 months of the preceding initial relapsing disease course [8]. Individual patient disease course is heterogeneous, and it is not clear what triggers conversion to SPMS [9], resulting in periods of diagnostic uncertainty and delays in SPMS diagnosis by approximately 3 to 4 years [6,10,11]. It has been suggested that the signs and symptoms of permanent neurological disability become evident as the functional capacity of the central nervous system to compensate for these tissue injuries is exhausted [9,12,13]. Therefore, there may be an optimal window of therapeutic opportunity, which—if missed—could leave only limited room to affect long-term outcomes in patients with MS [14]. Studies have reported that the onset of progression is early, with discrete and identifiable signs seen even at a disability status score of two or lower [2]. In many RRMS patients, silent accrual of disability progression independent of relapse activity has also been observed [15].

In previous research, physicians confirmed an unmet need for a tool that could be used in routine clinical practice to raise awareness and facilitate the systematic assessment of early signs of progression to SPMS. Physicians also expressed their preference for a validated digital solution producing an easy to interpret output [16]*.*

**Figure 1:**
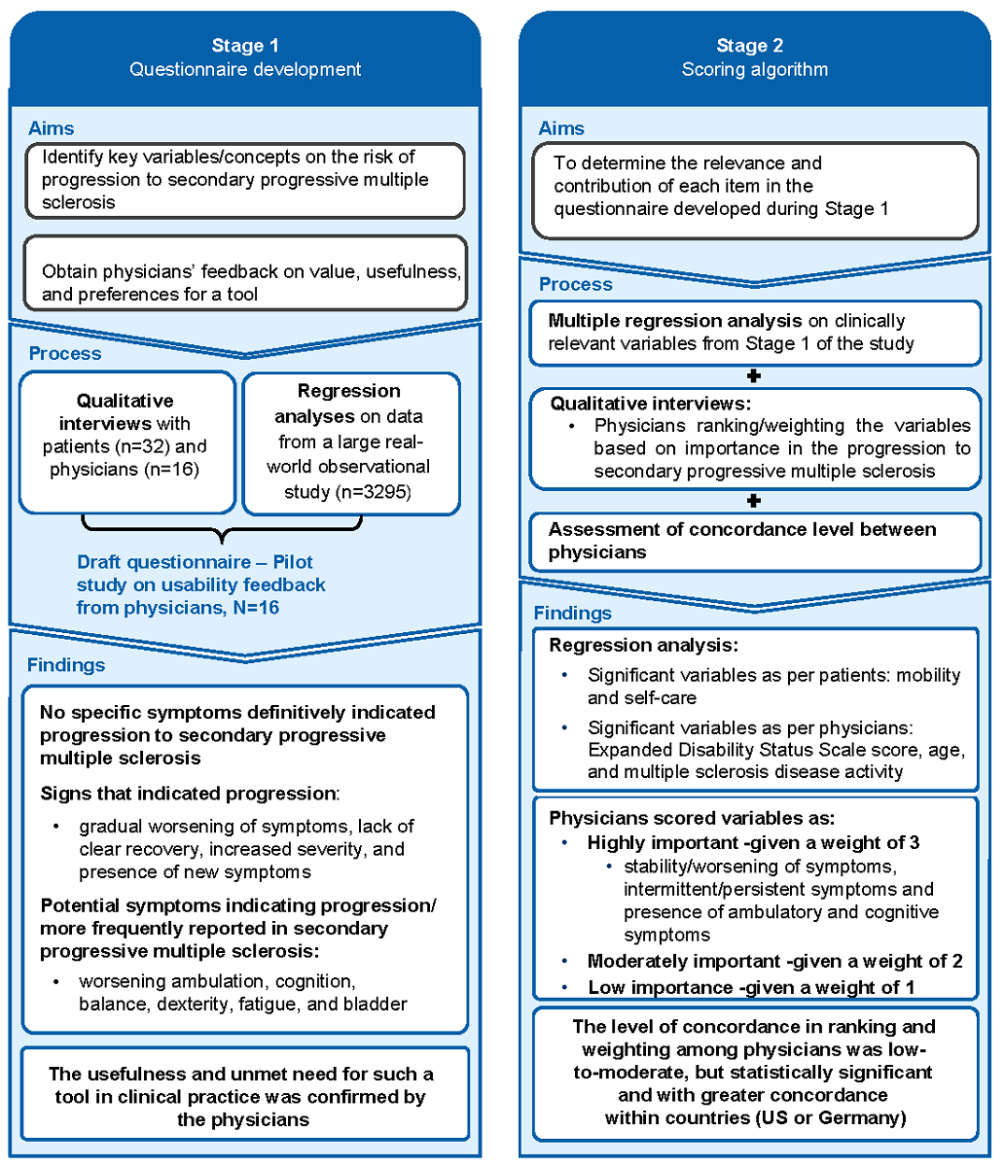
Overview of the development process of draft MSProDiscuss tool (a mixed methods approach).

With the preceding in mind, we developed MSProDiscuss, a digital tool to (1) facilitate physician-patient interaction in routine clinical practice; (2) support physicians in evaluating the early signs of progression in a structured, standardized manner based on a patient’s neurological history, the symptoms experienced, and how these affected various domains of the patient’s daily life in the past six months; and (3) help assess patient’s current level of progression. The content of this tool was developed using a mixed methods approach building on quantitative and qualitative assessments. Therefore, for the first time, both patients’ and physicians’ qualitative data were taken into consideration. The summary of the findings from stage 1 (development of the questionnaire) and stage 2 (scoring algorithm) of this comprehensive research is described in Figure 1 and has been published elsewhere ([16,17], also Tolley C et al, unpublished data, 2019). The tool captures different aspects related to disease progression that goes beyond the obvious signs of ambulatory impairment and provides an indication of the current likelihood of progression (Multimedia Appendix 1). In this paper, we evaluate the ability of the MSProDiscuss tool to differentiate between patients with RRMS transitioning to SPMS and those with SPMS to evaluate the reliability and validity of the draft tool and assess its usefulness in clinical practice.

## Methods

### Study Overview

Twenty physicians (seven from the United States, nine from Germany, and four from Canada) participated in this validation study. Physicians completed a Web-based draft version of the tool for up to 10 patients from their routine practice, comprising three to four patients each with a diagnosis of either RRMS or SPMS, or for patients that they suspected may be progressing to SPMS (“transitioning” patients). Physicians also completed a case report form (CRF) for each patient, which captured physician diagnosis (RRMS, SPMS, or transitioning) and other key clinical information. The order of the CRF and tool were alternated to minimize potential bias in tool completion. Physicians also provided information about their clinical experience by completing a physician CRF and provided their feedback on the content of the draft tool and usefulness of the tool in clinical practice by completing a usability questionnaire.

### Study Sample

A target of more than 150 patients was prespecified based on the planned receiver operating characteristic (ROC) analyses. The overall significance level for this study was set at .05, but this value was adjusted using a Bonferroni correction for the sample size calculation to take into account two testing procedures: SPMS versus not SPMS (RRMS and transitioning) and RRMS versus not RRMS (transitioning and SPMS). This led to a sample size that allowed detection of area under the ROC curve (ROC AUC) of at least 0.68 with 90% statistical power with a significance level of .025 [18].

### Physician Eligibility Criteria

Specialist neurologists, who were responsible for the care and management of at least five patients with MS per week, were included in this study once they provided written informed consent. The physician was required to be verbally fluent in their local language (either English or German).

### Patient Diagnosis Classification

As part of the CRF, physicians were required to specify each patient’s clinical diagnosis. RRMS was defined as having a confirmed diagnosis of RRMS according to the 2010 Revised McDonald Criteria [19]; SPMS was defined as a confirmed diagnosis of SPMS, indicated by a progressive increase in disability (of at least six months in duration) in the absence of relapses or independent of relapses *and* prior history of RRMS according to the 2010 Revised McDonald Criteria [19]. Transitioning was defined as a confirmed diagnosis of RRMS, according to the previously mentioned criteria; however, the physician believed that the patient may be progressing to SPMS based on recent clinical presentation. When possible, physicians completed the tool for an equal number of RRMS, SPMS, or transitioning patients.

### Overview of the Draft Tool

The draft tool consisted of three sections addressing disease activity, symptoms, and impact of these symptoms on patients’ daily living. Patients’ age, current Expanded Disability Status Scale (EDSS) score [20], and/or timed 25-foot walk test results were also collected. The draft tool provided a standardized total score by summing the raw score for each section and rescaling to a maximum possible score of 100 ([17] and also Tolley C et al, unpublished data, 2019).

For validation, two cut-off values were used to visualize the tool output: a score equal or above the upper cut-off indicated SPMS and values lower or equal to the chosen lower cut-off defined RRMS patients. The range between the upper and lower cut-offs indicated transitioning patients (RRMS patients showing early signs of progression but still not classified as SPMS by their treating physician). Following completion of the tool for a patient, an output screen visually displayed the standardized total score linked to a traffic light system, which was also used to obtain physician’s feedback on the usefulness of the tool in clinical practice.

### Statistical Analysis

SAS version 9 was used for all statistical analyses.

#### Disease Discrimination (Receiver Operator Characteristic Curves)

ROC curve analysis was used to evaluate the sensitivity (true positive rate) and specificity (true negative rate) of different cut-off values on the draft tool. The cut-off values informed the thresholds for which a patient would be classified as RRMS, SPMS, or transitioning. SPMS versus RRMS and transitioning patients were compared to obtain an upper cut-off for the tool, whereas RRMS versus transitioning and SPMS patients were compared to obtain a lower cut-off for the tool. Any value between the lower and upper thresholds was considered indicative of a patient possibly showing signs of progression (ie, in transition to SPMS). The cut-off values were estimated using Youden’s J index [21] and a sum of squares method [22], both placing equal weight on sensitivity and specificity (see details in Multimedia Appendix 2). Because EDSS is not always assessed in clinical practice and would not always be available for input into the tool, all ROC curve analyses were run twice, with and without EDSS score, to account for the impact of EDSS score on the overall performance of the tool.

#### Psychometric Properties

Two video vignettes depicting scenarios of mock patient-physician interactions were developed (one represented an SPMS patient [Multimedia Appendix 3] and one representing an RRMS patient [Multimedia Appendix 4]). These video vignettes were used to allow physicians to rate the same “patient.” Each physician completed a tool entry for each video vignette case study. Interrater reliability was assessed using the intraclass correlation coefficient (ICC_2,1_), with 0.75 or greater considered excellent interrater reliability, 0.40-0.75 as fair to good, and less than 0.40 as poor [23]. The validity of the tool was assessed by known-groups comparisons for patients who differed on EDSS score and physician disease diagnosis. The statistical significance of differences in scores between groups was calculated using two-sample *t* tests and the magnitude of the effect size estimates using Cohen *d*. Item correlations with physician diagnoses were assessed using Spearman correlations (*r*). For further details, refer to Multimedia Appendix 5.

#### Usability Analysis

Descriptive data were produced for physician responses to the usability questionnaire. Qualitative responses to the usability questionnaire were coded using thematic analysis methods on ATLAS.ti [24].

## Results

### Physician Demographics

A total of 20 physicians (all neurologists experienced in the treatment of MS) participated in the study. Neurologists reported seeing approximately 19 RRMS patients (range 5-54) and eight SPMS patients (range 1-25) in a week, with an average of 14.8 (SD 11.8) hours per week and an estimated average of 36.6% (SD 27.9%) of their monthly workload dedicated to MS patients (Multimedia Appendix 6). The neurologists worked across several settings, including private practice (70%, 14/20), academic settings (35%, 7/20), hospitals (30%, 6/20), primary care (10%, 2/20), and specialized MS clinics (5%, 1/20).

### Patient Demographics and Clinical Characteristics

In total, the neurologists completed the draft tool for 198 MS patients: 89 RRMS, 47 suspected to be transitioning, and 62 SPMS (according to their clinical diagnosis). Patients had a mean age of 44.8 (SD 12.8) years and a mean EDSS score of 4.0 (SD 1.7). The mean duration since RRMS diagnosis was 11.8 (SD 9.2) years—range 7.3 (SD 6.3) years (RRMS) to 17.3 (SD 10.1) years (SPMS)—and mean duration since SPMS diagnosis of 6.3 (SD 5.3) years (range <1-22 years; Table 1).

**Table 1:** Patient demographics and clinical characteristics by physician diagnosis (N=198).^a^

Patient characteristic		Physician diagnosis		
		RRMS (n=89)	Transitioning (n=47)	SPMS (n=62)
**Age (years)**				
	n	89	47	62
	Mean (SD)	38.1 (11.3)	46.2 (10.7)	53.4 (10.7)
	Median (range)	37 (19-66)	47 (28-68)	52 (34-78)
**EDSS score**				
	n	81	47	61
	Mean (SD)	2.6 (1.0)	4.3 (1.1)	5.6 (1.4)
	Median (range)	2 (0-7)^b,c^	4 (2-7)	6 (3-9)
**Patients with relapses in the past 6 months, n**				
	Yes	30	10	9
	No	59	37	53
**Duration since RRMS diagnosis (years)**				
	n	89	47	62
	Mean (SD)	7.3 (6.3)	13.2 (8.4)	17.3 (10.1)
	Median (range)^d^	5.0 (0-26)	11.3 (0-37)	15.8 (0-51)
**Duration since SPMS diagnosis (years)**				
	n	—	—	62
	Mean (SD)	—	—	6.3 (5.3)
	Median (range)^d^	—	—	5 (0-22)

^a^Physician diagnosis was collected from the patient case report form. EDSS: Expanded Disability Status Scale; RRMS: relapsing-remitting multiple sclerosis; SPMS: secondary progressive multiple sclerosis.

^b^EDSS=0 (n=1); EDSS=1 (n=0); EDSS=1.5 (n=1).

^c^Literature suggests that there are circumstances in which onset of progression to SPMS is early and identifiable at a DSS score of 2 or less [2].

^d^Minimum value of 0 indicates a duration of less than 12 months.

### Symptoms and Impacts

**Figure 2:**
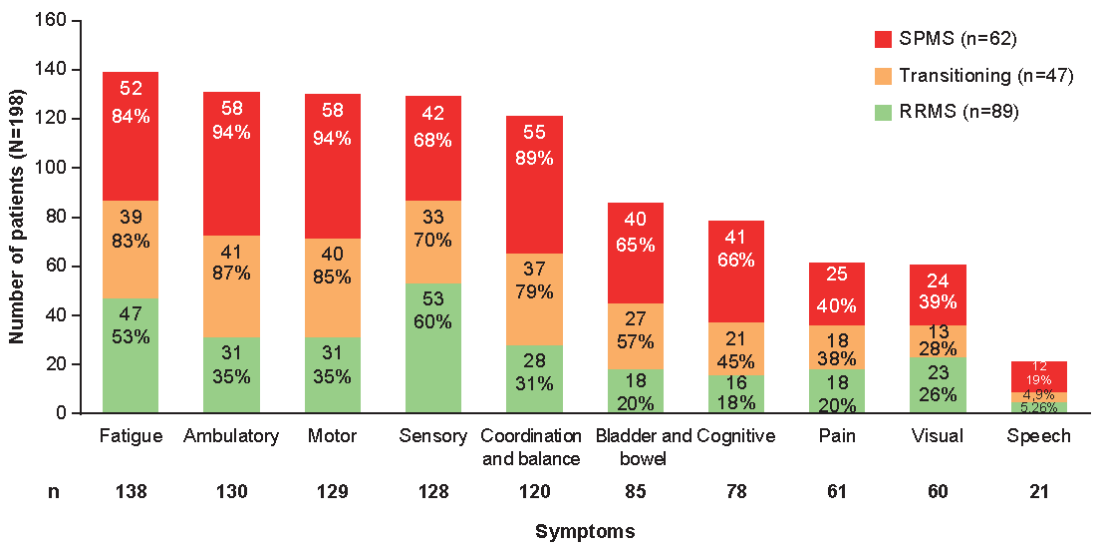
Number of patients experiencing each sign or symptom by physician diagnosis. RRMS: relapsing-remitting multiple sclerosis; SPMS: secondary progressive multiple sclerosis.

According to the data entered into the draft tool (Figure 2), the symptoms most frequently experienced by MS patients were fatigue (70%, 138/198), ambulatory (66%, 130/198), motor (65%, 129/198), sensory (65%, 128/198), and problems with coordination and balance (61%, 120/198). All symptoms were more frequent in SPMS and transitioning patients than RRMS, with pronounced differences observed for cognitive symptoms (66%, 41/62 and 45%, 21/47 versus 18%, 16/89), bowel and bladder symptoms (65%, 40/62 and 57%, 27/47 versus 20%, 18/89), ambulatory and motor symptoms (94%, 58/62 SPMS patients for both and 87%, 41/47 and 85%, 40/47 transitioning patients versus 35%, 31/89 RRMS patients for both), and coordination and balance (89%, 55/62 and 79%, 37/47 versus 31%, 28/89).

**Figure 3:**
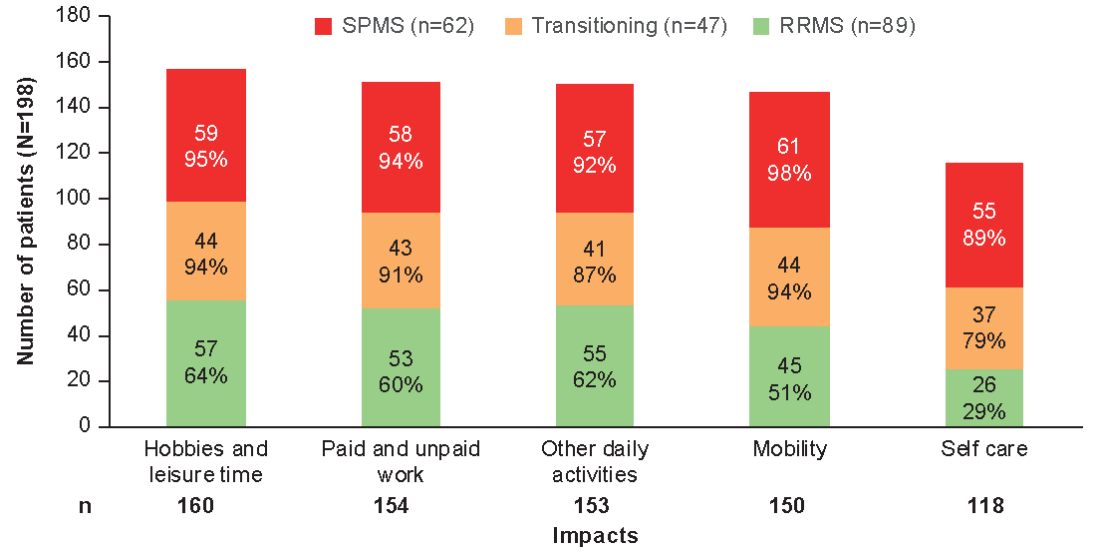
Number of patients experiencing each impact by physician diagnosis. RRMS: relapsing-remitting multiple sclerosis; SPMS: secondary progressive multiple sclerosis.

The impact of symptoms was experienced in all domains of patients’ daily life (Figure 3). SPMS and transitioning patients experienced greater impacts across all domains, with self-care items showing the largest difference (89%, 55/62 and 79%, 37/47 versus 29%, 26/89 for RRMS patients) followed by mobility (98%, 61/62 and 94%, 44/47 versus 51%, 45/89). Additionally, the impacts were more severe for SPMS and transitioning patients compared with RRMS patients (Multimedia Appendix 7).

### Performance of the Scoring Algorithm for Draft Tool

**Figure 4:**
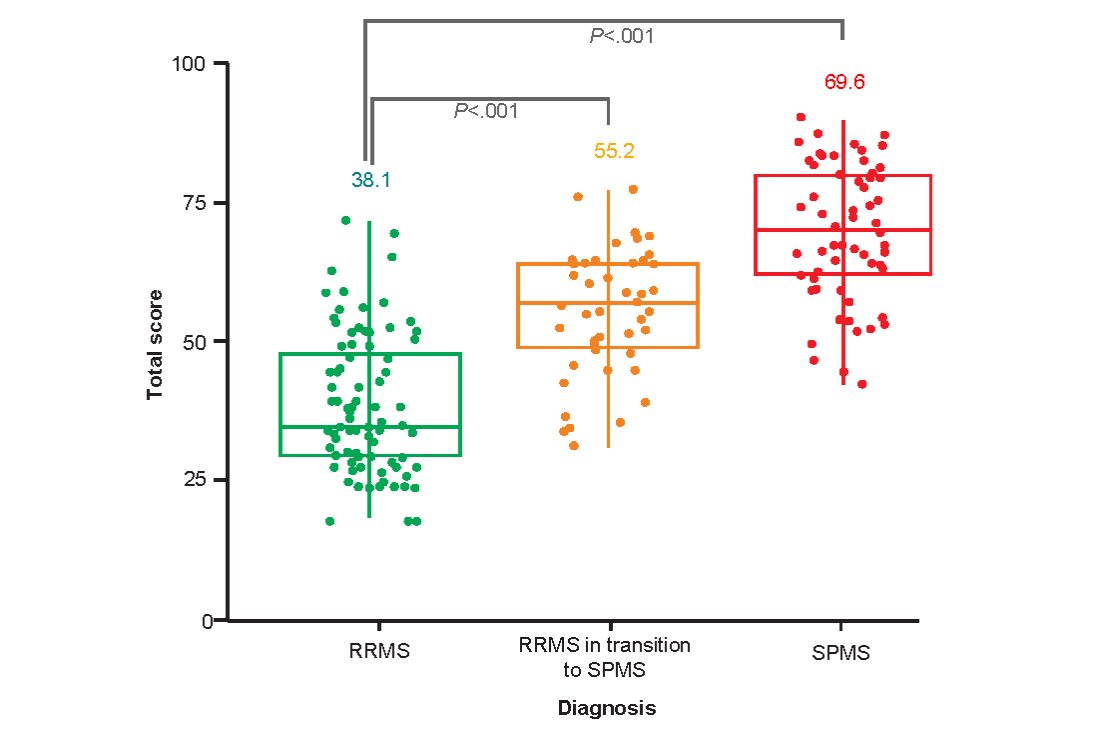
Distribution of total scores according to physician MS diagnosis. The values within the figure are mean scores. *P* values are for between groups comparison versus RRMS. RRMS: relapsing-remitting multiple sclerosis; SPMS: secondary progressive multiple sclerosis.

Patients with a physician diagnosis of SPMS scored higher (mean 69.6, SD 12.0) than those patients suspected to be in transition to SPMS (mean 55.2, SD 11.1) and those with a physician diagnosis of RRMS (mean 38.1, SD 12.5, *P*<.001; Figure 4).

### Disease Discrimination (Receiver Operator Characteristic Curves)

**Figure 5:**
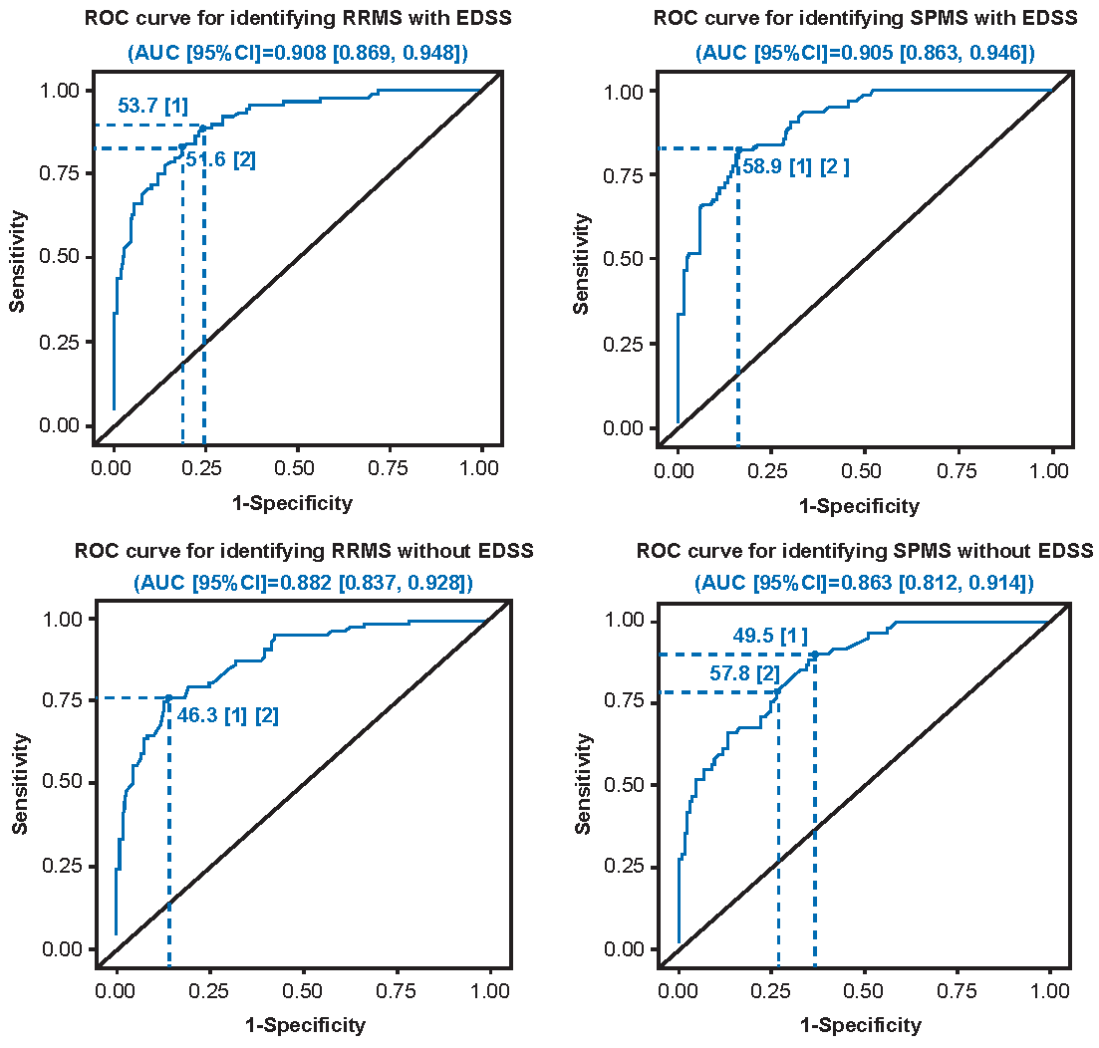
Receiver operator characteristic (ROC) curves and cut-off points identified by Youden’s J index and sum of squares. AUC: area under the curve; EDSS: Expanded Disability Status Scale; RRMS: relapsing-remitting multiple sclerosis; SPMS: secondary progressive multiple sclerosis.

Youden’s J index and the sum of squares method estimated the optimal cut-off values, with equal weight for sensitivity and specificity (Table 2, Figure 5). The ROC curves, with and without EDSS and for all comparisons, had moderate to high [18] AUC values (0.86-0.91). When the ROC analysis was initially run with EDSS included, the lower cut-off score was estimated as 51.6 (sum of squares) or 53.7 (Youden’s J), whereas both methods estimated an upper cut-off of 58.9. Sensitivity exceeded 0.82 and specificity exceeded 0.76 for all estimated cut-offs. With the ROC analysis without EDSS, the lower cut-off was estimated as 46.3 through both methods, whereas an upper cut-off was 57.8 (sum of squares) or 49.5 (Youden’s J). Sensitivity exceeded 0.76 for all estimated cut-offs values; however, the specificity of the 49.5 upper cut-off was markedly lower in comparison to the alternative estimate of 57.8 (0.63 versus 0.74). Sensitivity and specificity remained high when the selected cut-off points obtained by excluding EDSS (≤46.3 and ≥57.8) were applied to the algorithm including EDSS (Table 3, Multimedia Appendix 8).

**Table 2:** Cut-off points identified using the Youden’s J index and sum of squares methods.^a^

Method			Cut-off	AUC (95% CI)	Sensitivity	Specificity
**SPMS versus transitioning and RRMS (upper cut-off)**						
	**With EDSS**					
		Youden’s J index, sum of squares	58.9	0.91 (0.86–0.95)	0.82	0.84
	**Without EDSS**			0.86 (0.81–0.91)		
		Sum of squares	57.8		0.79	0.74
		Youden’s J index	49.5		0.90	0.63
**RRMS versus transitioning and SPMS (lower cut-off)**						
	**With EDSS**			0.91 (0.87–0.95)		
		Sum of squares	51.6		0.83	0.82
		Youden’s J index	53.7		0.89	0.76
	**Without EDSS**					
		Youden’s J index, sum of squares	46.3	0.88 (0.84–0.93)	0.76	0.86

^a^AUC: area under the curve; EDSS: Expanded Disability Status Scale; RRMS: relapsing-remitting multiple sclerosis; SPMS: secondary progressive multiple sclerosis.

**Table 3:** Sensitivity and specificity by applying the same cut-off points for the draft algorithm with and without EDSS.^a^

Draft algorithm		Cut-off	Sensitivity	Specificity
**SPMS versus transitioning and RRMS (upper cut-off)**				
	With EDSS	>57.8	0.82	0.81
	Without EDSS		0.79	0.74
**RRMS versus transitioning and SPMS (lower cut-off)**				
	With EDSS	<46.3	0.72	0.89
	Without EDSS		0.76	0.86

^a^EDSS: Expanded Disability Status Scale; RRMS: relapsing-remitting multiple sclerosis; SPMS: secondary progressive multiple sclerosis.

### Psychometric Properties

#### Interrater Reliability

The total score for the draft tool demonstrated excellent interrater reliability (ICC 0.950, 95% CI 0.772-1.000). The ICC was good for the disease activity section (ICC 0.852, 95% CI 0.504-1.000) and the symptoms section (ICC 0.869, 95% CI 0.541-1.000), and close to fair (ICC 0.391, 95% CI 0.073-0.999) for the impacts section.

#### Known-Groups Validity

A statistically significant difference was observed in the total score between EDSS groups and physician diagnosis groups (*P*<.001), indicating that the total score could discriminate between EDSS and diagnosis subgroups (Multimedia Appendix 9). Known-groups findings for section scores were similar, with a statistically significant difference observed between EDSS and physician diagnosis subgroups (Multimedia Appendix 10). The mean total score was higher for patients with a worse EDSS score (>4.5 and <9.5 versus ≥1 and ≤4.5; mean 70.2, SD 10.6 versus 43.2, SD 14.0, *P*<.001).

#### Item Correlations

The majority of items included in the draft tool showed strong (*r*>.7) to moderate (*r*>.5) correlations with total score and physician diagnosis (Multimedia Appendix 11).

#### Usability Testing

The mean time to complete the draft tool was 2.16 minutes per patient (n=83; median 1.59, range 0.48-6.58). Findings from the usability testing are provided in Table 4. All neurologists completed the usability questionnaire and provided feedback on the various aspects of the tool, with 17 of 20 neurologists (85%) expressing that it would be feasible to implement the tool in clinical practice because these data are collected typically in clinical practice. All neurologists confirmed the items included were relevant to progression; only two neurologists suggested potential inclusion of additional items, such as anxiety and depression, and to assess whether symptoms were new. Overall, they were satisfied by the time taken to complete the tool and found the traffic light signal related to the level of progression clear and useful.

**Table 4:** Findings from the usability testing of the draft tool (N=20).

Usability statements		n (%)
Items relevant to identifying progression to SPMS^a^		20 (100)
Typically collect tool data in clinical practice		18 (90)
**Information missing from the tool**		
	Anxiety and depression	2 (10)
	New symptoms	2 (10)
Time to complete is satisfactory		17 (85)
Traffic light style output is useful and clear		16 (80)
Feasible to implement tool in clinical practice		17 (85)
Open to using the tool as an additional independent evaluation to complement neurological assessment		17 (85)

^a^SPMS: secondary progressive multiple sclerosis.

## Discussion

The MSProDiscuss tool was able to differentiate between RRMS and SPMS patients, with the highest scores seen in patients with a diagnosis of SPMS. The sensitivity for SPMS was consistently around 80% (true positive rate) and specificity (true negative rate) for RRMS above 86%. Overall, the draft tool demonstrated excellent interrater reliability, and good evidence of construct validity using the known-groups method. The neurologists supported the implementation and usefulness of the tool for clinical practice. The items in the draft tool were considered relevant and are typically collected in this setting; hence, they do not represent an additional burden to the clinical practice.

Disease evolution is highly variable in MS, and progression to SPMS is a key milestone for patients. The use of tools supporting the real-time evaluation of early signs of MS progression for use in daily practice is currently an unmet need. A number of studies have investigated predictors of SPMS; however, only a few reported on tools to predict SPMS progression. These tools have been derived using only quantitative empirical assessments of different registry-based databases [11,25] or are intended mainly for use in research studies and may not be applicable for routine clinical practice [26]. These algorithms and nomograms are data-derived and rely on available data in the respective databases [8]. However, qualitative assessments are important instruments that can provide additional insights on relevant aspects assessed in daily clinical practice that are not routinely collected in registries.

MSProDiscuss was developed using a rigorous mixed methods approach, which incorporated a regression analysis of data from a large observational study and qualitative interviews with patients and physicians. Items included in the tool were previously identified as relevant and suggestive of progressive disease ([16],17] and also Tolley C et al, unpublished data, 2019). These studies highlighted the importance of assessing symptoms that go beyond the obvious signs of physical worsening, such as cognitive impairment, which is known to occur very early in the disease course, even before physical disability accrual, and are predictive of further progression. Furthermore, persistent worsening of any symptom emerged as one of the most important indicators of progression to SPMS, even more than a specific symptom itself.

MSProDiscuss is an easy to use physician-completed digital tool intended to facilitate physician-patient dialog in assessing the subtle signs suggestive of disease progression by systematically evaluating and recalling relevant information from patient clinical history, symptoms, and impacts on daily activities experienced over the past six months. Such variables are often assessed in routine clinical practice, but might not be systematically recorded.

### Principal Findings

In this study, we validated the draft tool and algorithm developed based on the findings from previous studies ([16,17] and also Tolley C et al, unpublished data, 2019), and identified the cut-off values for optimal discrimination between patients with RRMS and SPMS. Incorporating two cut-off values in the tools’ algorithm (eg, an upper cut-off for SPMS and a lower cut-off for RRMS) allowed us to define a separate “transitioning patients” group possibly showing signs of progression. It is essential to identify the subtle signs that are indicative of progression early to maximize the therapeutic window of opportunity to affect the course of the disease [14]. Recently “silent” insidious progression has been described in many early RRMS patients [15]. However, these patients remained classified as having relapsing MS and, as a consequence, might not be optimally managed for the disability progression [15].

The impact and severity of symptoms experienced by transitioning and SPMS patients were clearly different compared with patients with RRMS. All symptoms assessed were more frequently reported in SPMS and transitioning patients compared with RRMS patients. Specifically, symptoms related to ambulatory, motor, coordination and balance, bladder and bowel, and cognition were approximately 2.5 to 3.5 times more frequent in SPMS and transitioning patients compared with RRMS patients. These results are consistent with the findings from the qualitative assessment in our previous pilot study ([16,17] and also Tolley C et al, unpublished data, 2019). The vast majority of SPMS and transitioning patients (80%-98% vs 50%-60% of RRMS patients) were affected across all domains of patients’ daily life, with the most pronounced difference versus RRMS patients in the domain of self-care and mobility. Nevertheless, two of three patients with RRMS experienced impacts on hobbies and leisure time, paid and unpaid work, and other daily activities, confirming the serious impact of this disease on patients’ lives also during early RRMS stage. 

The ROC analysis confirmed that the draft tool was able to discriminate between SPMS and RRMS patients with high sensitivity and specificity. Although a stronger performance was observed when EDSS was included in the draft algorithm (AUC 0.905-0.908), the tool also maintained good performance in the absence of EDSS (AUC 0.863-0.882). Cut-offs excluding EDSS were considered appropriate for the final validated tool, as it is intended for use in clinical practice where EDSS might not be routinely assessed. Sensitivity for SPMS was consistently around 80% (true positive rate) and specificity (true negative rate) for RRMS above 86%. The excellent interrater reliability and good evidence of construct validity suggest that the items included in this tool are of relevance to assess early signs of progression. The average time to complete the tool was approximately 2 minutes, and in the usability testing, neurologists supported the implementation and usefulness of the tool for clinical practice.

### Study Limitations and Future Outlook

Some of the analyses were based on physician diagnosis; however, they also completed the tool, which may have introduced some reporting bias. To overcome this potential bias, the order of completion of the patient CRF, including the physician diagnosis and the draft tool, were alternated. Also, neurologists in this study were all well-experienced in diagnosing and managing MS patients; hence, they did not rely on the tool for their diagnosis of SPMS. Importantly, the tool was validated not only against SPMS but also RRMS and patients in transition. Moreover, patients and physicians from different countries were involved (including the United States, Canada, and Germany) to reduce potential bias due to differences in health care systems and approaches adopted to diagnose SPMS, which became evident from previous research ([16,17] and also Tolley C et al, unpublished data, 2019).

The MSProDiscuss tool is now included in a large real-world observational study in Germany (PANGAEA 2.0 [27,28]); 1000 MS patients will be followed-up for two years and evaluated by the tool every six months. This study will provide useful insights on the utility of the tool in the longitudinal monitoring of symptoms and impacts and correlation with other clinical measures in routine clinical practice. The tool will also be assessed in terms of longitudinal validity in which changes in scores are compared with change in diagnosis from RRMS to SPMS. The tool could be personalized for country- or clinic-specific requirements; sensitivity and specificity could be further increased by adding biomarkers of interest. Furthermore, a patient-completed version of the tool, to serve as a communication aid, and a nurse-completed version to help physicians manage time pressure, could be valuable in clinical practice.

### Conclusion

The aim of MSProDiscuss is to facilitate physician-patient conversation by allowing a comprehensive, but simple, standardized assessment of the patient’s current disease status and level of progression. In this validation study, the MSProDiscuss tool demonstrated its ability to differentiate between patients with RRMS and SPMS with high sensitivity and specificity, with or without EDSS. Thus, the tool will help sensitize for early, subtle signs suggestive of MS disease progression in daily practice. The tool also supports the evaluation of transitioning patients who have not yet converted to SPMS and who might benefit most from optimized early interventions that slow disability accumulation. Evidence from this study suggests the tool may be useful in clinical practice for a more informed physician-patient discussion supporting the successful management of MS.

The final validated MSProDiscuss tool can be accessed on the Neuro-Compass website [29].

## Appendix

Multimedia Appendix 1 MSProDiscuss: Screenshots of the tool.

Multimedia Appendix 2 Statistical analysis.

Multimedia Appendix 3 Video vignette-SPMS patient.

Multimedia Appendix 4 Video vignette-RRMS patient.

Multimedia Appendix 5 Psychometric properties.

Multimedia Appendix 6 Physician characteristics.

Multimedia Appendix 7 Number of patients experiencing each impact, by severity.

Multimedia Appendix 8 ROC curves for selected cut-off points (applying same cut-offs to algorithm including and excluding EDSS.

Multimedia Appendix 9 Known-groups comparisons for total score.

Multimedia Appendix 10 Known-groups comparisons for section scores.

Multimedia Appendix 11 Item correlations with total score and item correlations with physician diagnosis.

## Abbreviations

AUC: area under the curve
CRF: case report form
EDSS: Expanded Disability Status Scale
ICC: interclass correlation coefficient
MS: multiple sclerosis
ROC: receiver operating characteristic
RRMS: relapsing-remitting multiple sclerosis
SPMS: secondary progressive multiple sclerosis
