# Supplementary material for: A Physician-Completed Digital Tool for Evaluating Disease Progression (Multiple Sclerosis Progression Discussion Tool): Validation Study
Source: J Med Internet Res. 2020 Feb 12;22(2):e16932. doi: 10.2196/16932 (PMC7055760; doi:10.2196/16932)
Supplement: Multimedia Appendix 1 [file jmir_v22i2e16932_app1.docx]

# Multimedia Appendix 1. MSProDiscuss^TM^: Screenshots

Screenshot 1: Patient data: age, clinical and MRI features in the past 6 months


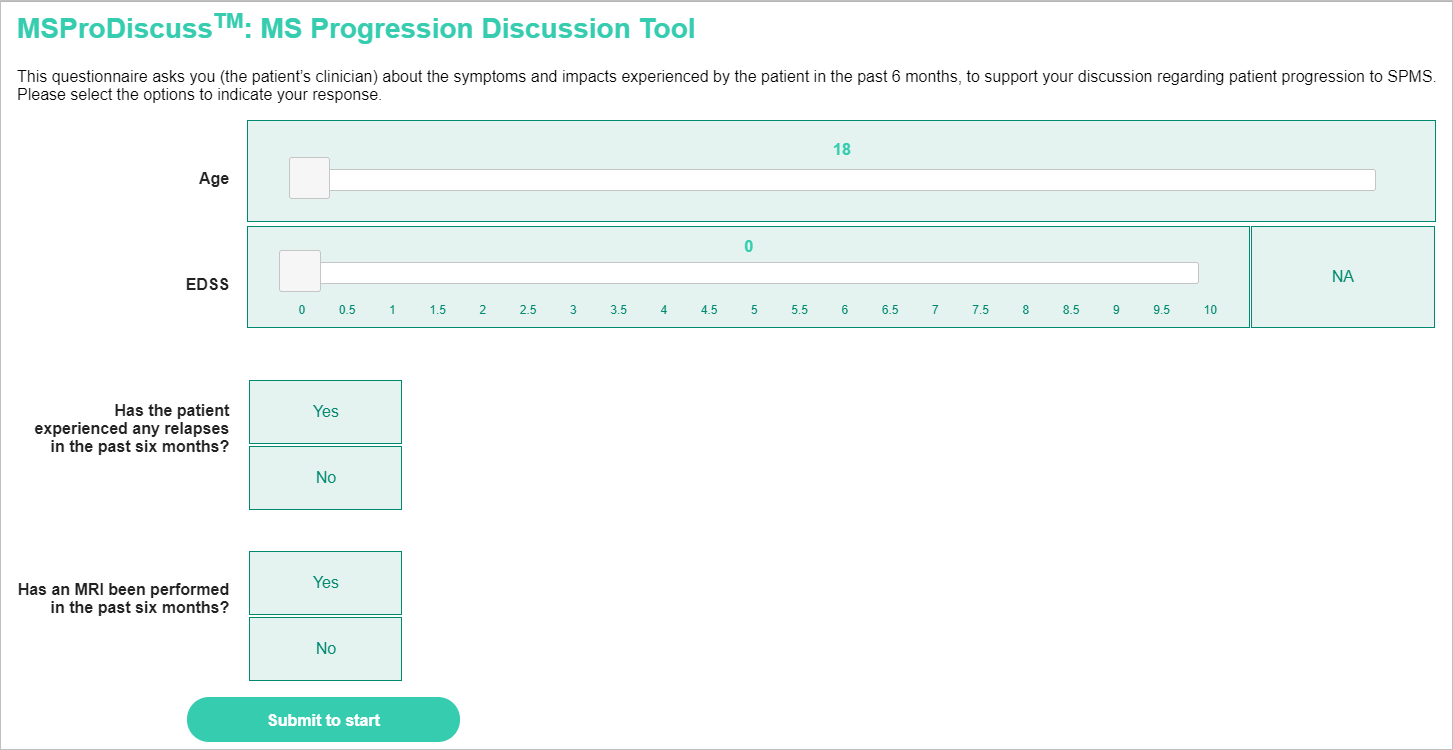


Screenshot 2: Symptoms experienced in the past 6 months


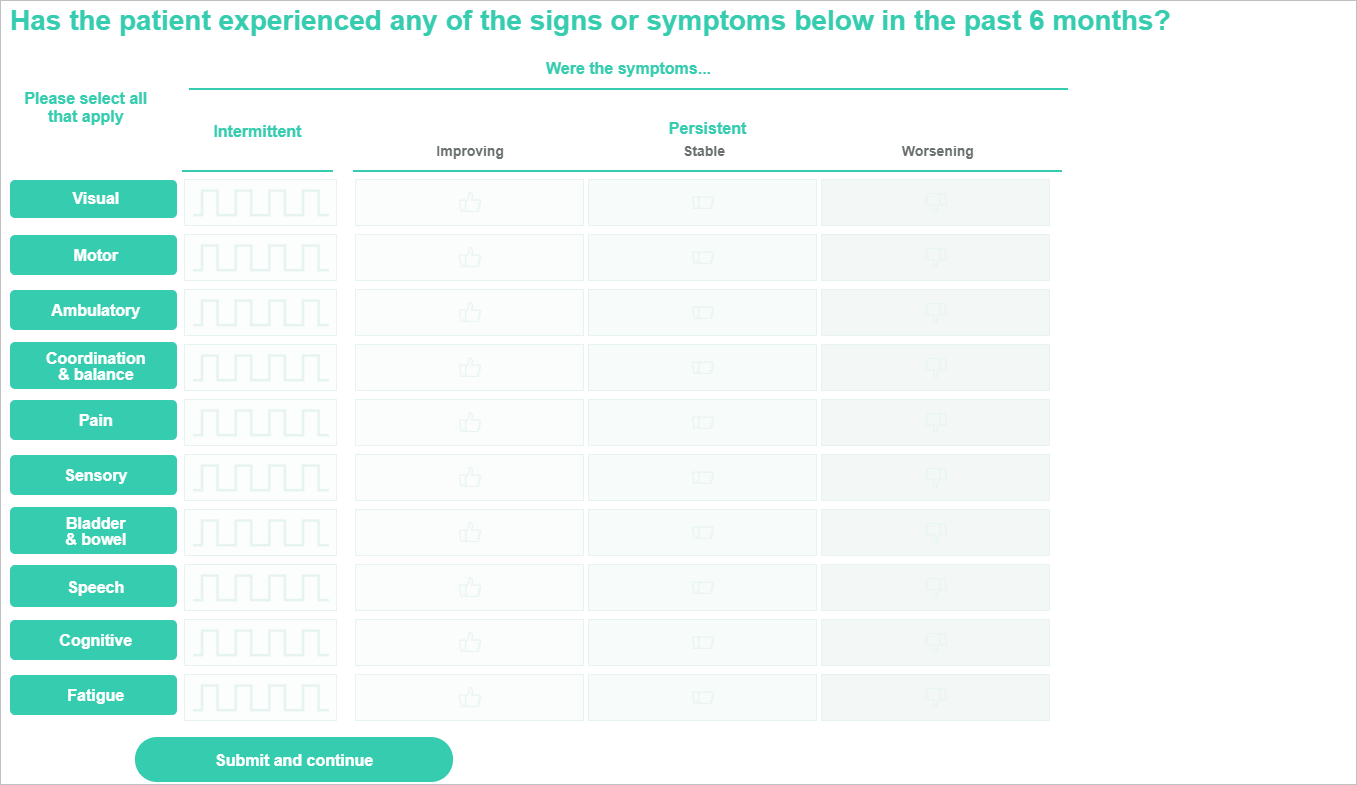


Screenshot 3: Impact of the symptoms experience in the last 6 months


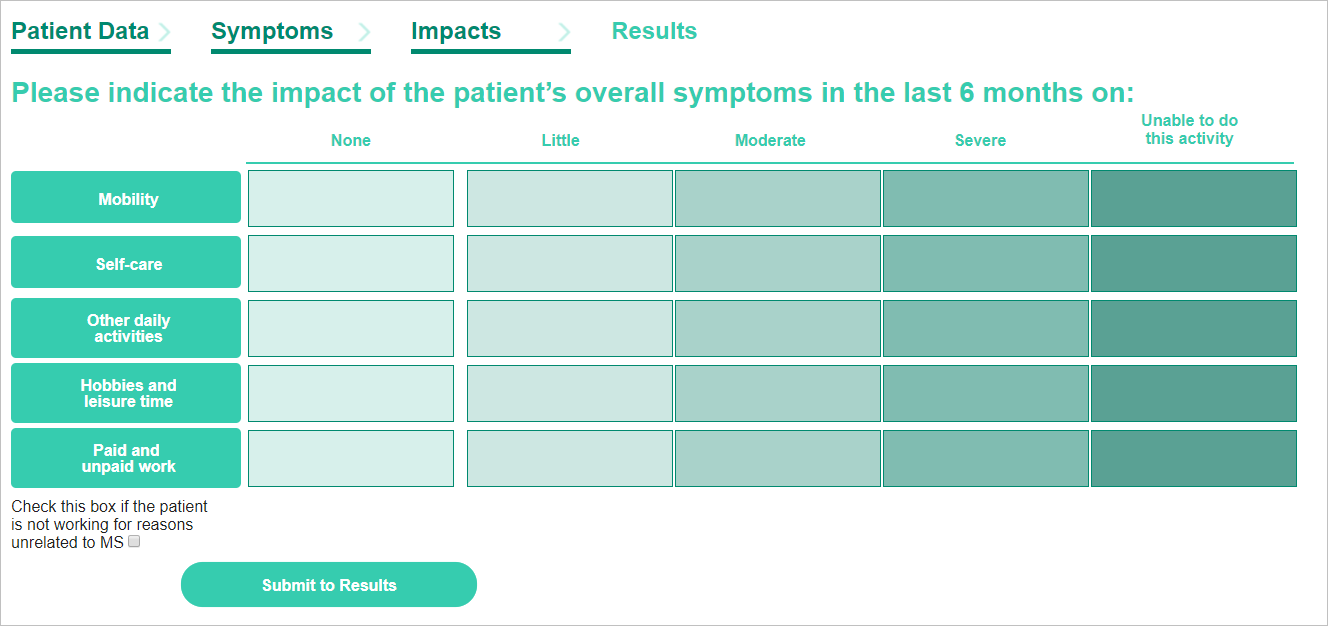


Output Screen: Traffic light system linked visual display and level of progression


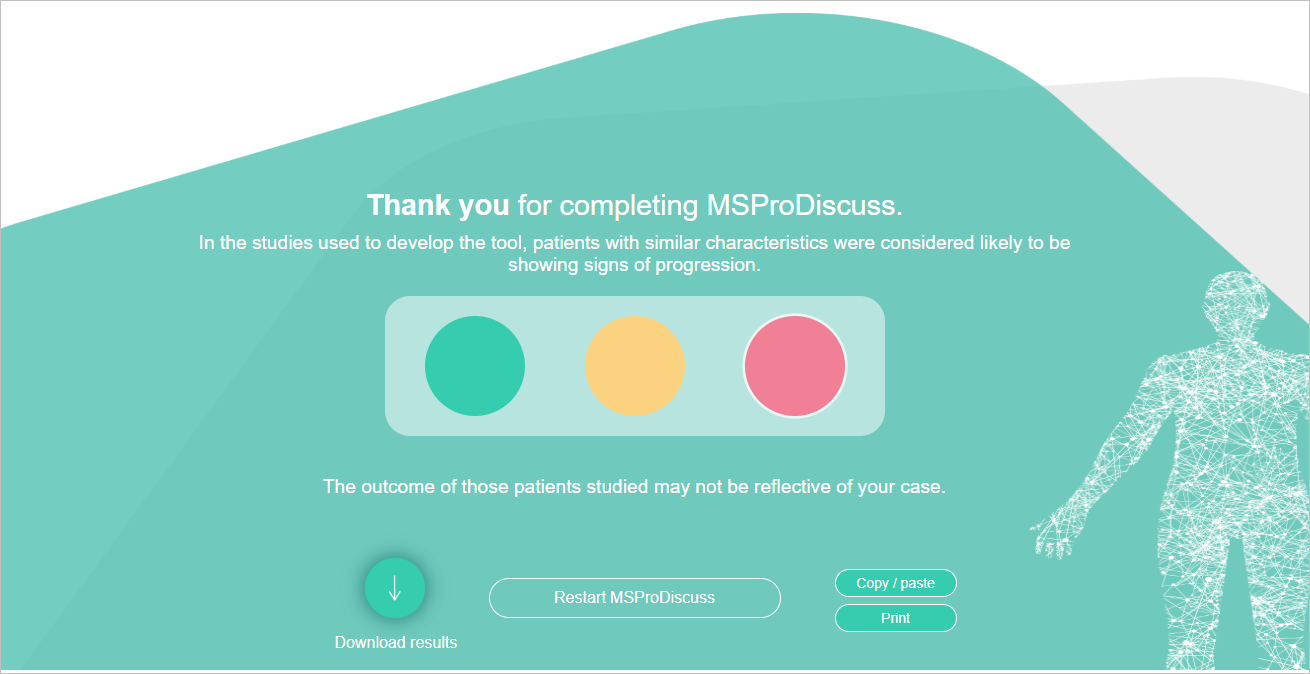


INFO: The MS Progression Discussion Tool is a clinician-completed tool for use by healthcare professionals in clinical practice to educate and sensitize about the risk of progressing from RRMS to SPMS and facilitate physician-patient interaction. This tool is for educational and discussion purposes only. The tool uses information from patient symptoms and impacts experienced in the past 6 months to generate a traffic light system that complements physician discussion regarding patient progression to SPMS. It was developed based on qualitative research with clinicians and patients and empirical assessments of real-world evidence. It has been pilot-tested and validated with clinicians in the real world.

This tool does not provide medical advice, diagnosis, prediction, prognosis, or treatment. The tool and its content are being provided for general information purposes only. Any medical advice, diagnosis or treatment should be made by the appropriate healthcare professional. The development of this tool has been funded by Novartis Pharma.
