# Supplementary material for: A Physician-Completed Digital Tool for Evaluating Disease Progression (Multiple Sclerosis Progression Discussion Tool): Validation Study
Source: J Med Internet Res. 2020 Feb 12;22(2):e16932. doi: 10.2196/16932 (PMC7055760; doi:10.2196/16932)
Supplement: Multimedia Appendix 2 [file jmir_v22i2e16932_app2.docx]

### Statistical analysis

Two different statistical methods were considered to determine the cut-off values that placed equal weight on sensitivity and specificity, Youden’s J index and the sum of squares.

Youden’s J index: Max[sensitivity+specificity−1], i.e. maximize the sum of sensitivity and specificity.

Sum of squares: Min[(1−sensitivity)^2^+(1−specificity)^2^], i.e. the point in the Receiver Operating Characteristic (ROC) space that minimizes the sum of squares, which is equivalent to choosing the cut point closest to the top-left corner in the ROC curve.
