# Supplementary material for: A Physician-Completed Digital Tool for Evaluating Disease Progression (Multiple Sclerosis Progression Discussion Tool): Validation Study
Source: J Med Internet Res. 2020 Feb 12;22(2):e16932. doi: 10.2196/16932 (PMC7055760; doi:10.2196/16932)
Supplement: Multimedia Appendix 5 [file jmir_v22i2e16932_app5.docx]

### Psychometric properties

#### **Reliability**

Interrater reliability is measured to address concerns as to whether scores are consistent when different raters use the instrument to rate the same individual. Interrater reliability was evaluated to ensure that subjective bias is reduced as much as possible, i.e. that clinicians are completing the tool consistently. Two video vignettes, which included subtitles, depicting scenarios of mock patient-physician interactions, one representing a secondary progressive multiple sclerosis (SPMS) patient and one representing a relapsing-remitting MS (RRMS) patient, were developed. Interrater reliability was assessed using these video case vignettes, which allowed physicians to rate the same patient case study. Each physician completed two tool entries, corresponding to each of the two video case vignettes. The intraclass correlation coefficient was used, with >0.75 considered “excellent” inter-rater reliability, 0.40-0.75 as “fair to good”, and <0.40 as “poor”.

#### **Validity**

Known-groups comparisons evaluate the ability of a measure to differentiate between variables hypothesized to influence the construct of interest (i.e. SPMS diagnosis). Known-groups analysis was conducted for patients who differed on Expanded Disability Status Scale (EDSS) score and physician disease diagnosis. Analyses were only performed in instances where there were at least 20 patients in each category. The statistical significance of differences in scores between groups was calculated using two-sample t-tests. The magnitude of these differences was considered using effect size estimates (Cohen’s *d*). The mean and median total scores for each known-group are presented to allow for comparisons. Cohen’s *d* was used to measure the differences between the mean scores, with a *d*=0.2 considered a 'small' effect size, 0.5 a 'medium' effect size, and 0.8 a 'large' effect size. A 'large' value of *d* implies that the difference between the two groups is large and consistent enough to be important.

Item-total score correlations provide insight into which items correlate most strongly with the total score. Spearman correlations between each item and the sum of all items included in the total score were produced. Item correlations with physician diagnosis provide insight into which items correlate most strongly with the diagnosis of patients by physicians. Spearman correlations between each item and physician diagnosis were produced.
