# Supplementary material for: A Physician-Completed Digital Tool for Evaluating Disease Progression (Multiple Sclerosis Progression Discussion Tool): Validation Study
Source: J Med Internet Res. 2020 Feb 12;22(2):e16932. doi: 10.2196/16932 (PMC7055760; doi:10.2196/16932)
Supplement: Multimedia Appendix 6 [file jmir_v22i2e16932_app6.docx]

# Table: Physician characteristics

| Demographic characteristic | Physicians (N=20) |
| --- | --- |
| Gender, n (%) | |
| Male | 14 (70.0) |
| Female | 6 (30.0) |
| Age | |
| Mean [SD] (range) | 53.7 [6.1] (41-63) |
| Current role, n (%) | |
| Neurologist | 20 (100.0) |
| Position/professional title, n (%) | |
| MD/Dr | 11 (55.0) |
| Neurologist | 4 (20.0) |
| Professor | 3 (15.0) |
| MS Specialist | 1 (5.0) |
| Consultant | 1 (5.0) |
| Years in role current role | |
| Mean [SD] (range) | 20.3 [8.0] (9.0-35.0) |
| Current work setting, n (%)* | |
| Private practice | 14 (70.0) |
| Academic (e.g. university or college) | 7 (35.0) |
| Hospital based care | 6 (30.0) |
| Primary care | 2 (10.0) |
| MS clinic | 1 (5.0%) |
| Number of RRMS patients seen in a typical week | |
| Mean [SD] (range) | 19.3 [13.9] (5-54) |
| Number of SPMS/ suspected SPMS patients in a typical week | |
| Mean [SD] (range) | 7.5 [7.0] (1-25) |
| Percentage of monthly workload dedicated to MS | |
| Mean [SD] (range) | 36.6 [27.9] (8.0-80.0) |
| Number of hours dedicated to MS patients per week | |
| Mean [SD] (range) | 14.8 [11.8] (3-45) |
| *Physicians could select more than one option | |

n, number of physicians

RRMS, relapsing–remitting multiple sclerosis; SPMS, secondary progressive multiple sclerosis
