# Supplementary material for: A Physician-Completed Digital Tool for Evaluating Disease Progression (Multiple Sclerosis Progression Discussion Tool): Validation Study
Source: J Med Internet Res. 2020 Feb 12;22(2):e16932. doi: 10.2196/16932 (PMC7055760; doi:10.2196/16932)
Supplement: Multimedia Appendix 7 [file jmir_v22i2e16932_app7.docx]

# Figure: Number of patients experiencing each impact, by severity


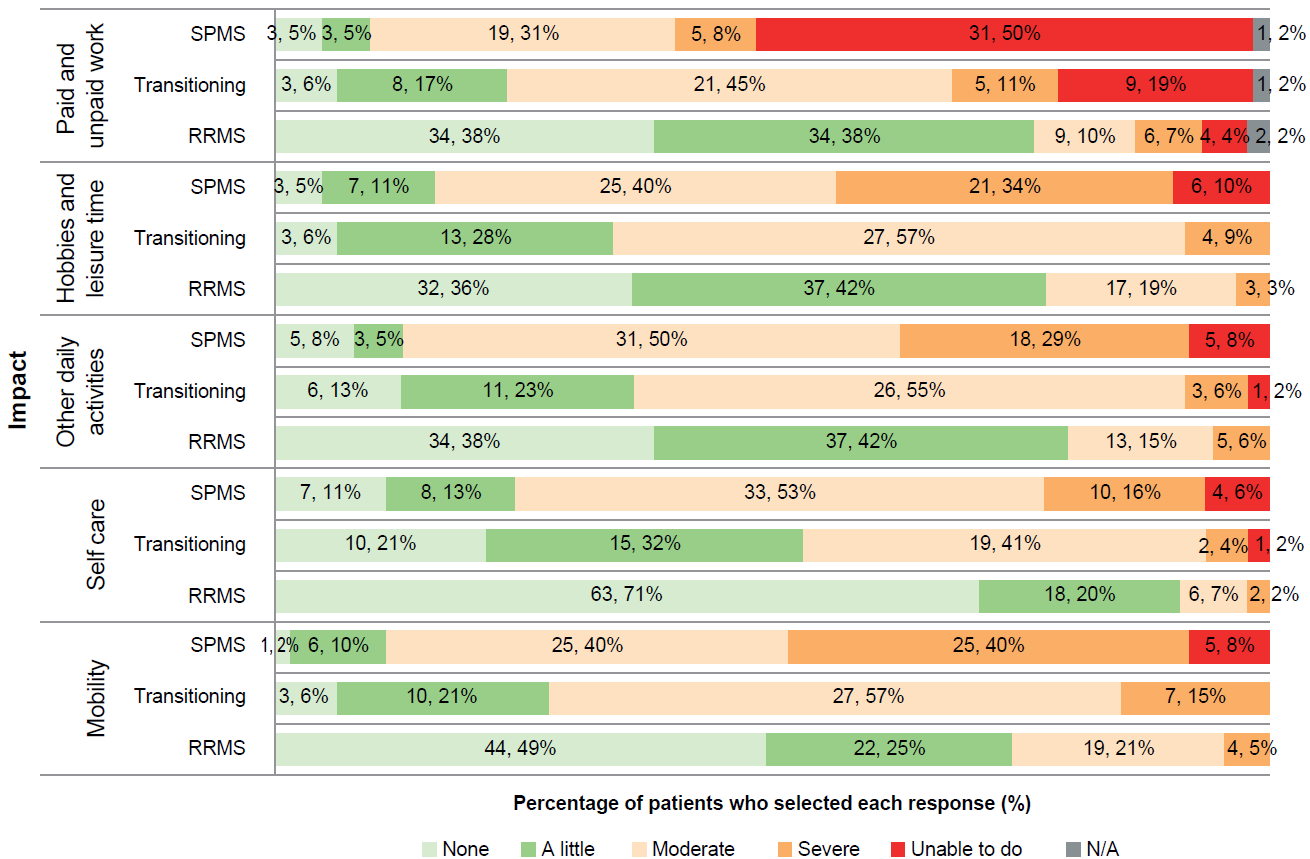


Numbers on the bars represent number, % of patients who selected each response

N/A, not available; RRMS, relapsing–remitting multiple sclerosis; SPMS, secondary progressive multiple sclerosis
