# Supplementary material for: A Physician-Completed Digital Tool for Evaluating Disease Progression (Multiple Sclerosis Progression Discussion Tool): Validation Study
Source: J Med Internet Res. 2020 Feb 12;22(2):e16932. doi: 10.2196/16932 (PMC7055760; doi:10.2196/16932)
Supplement: Multimedia Appendix 8 [file jmir_v22i2e16932_app8.docx]

# Figure: ROC curves for selected cut-off points (applying same cut-offs to algorithm including and excluding EDSS


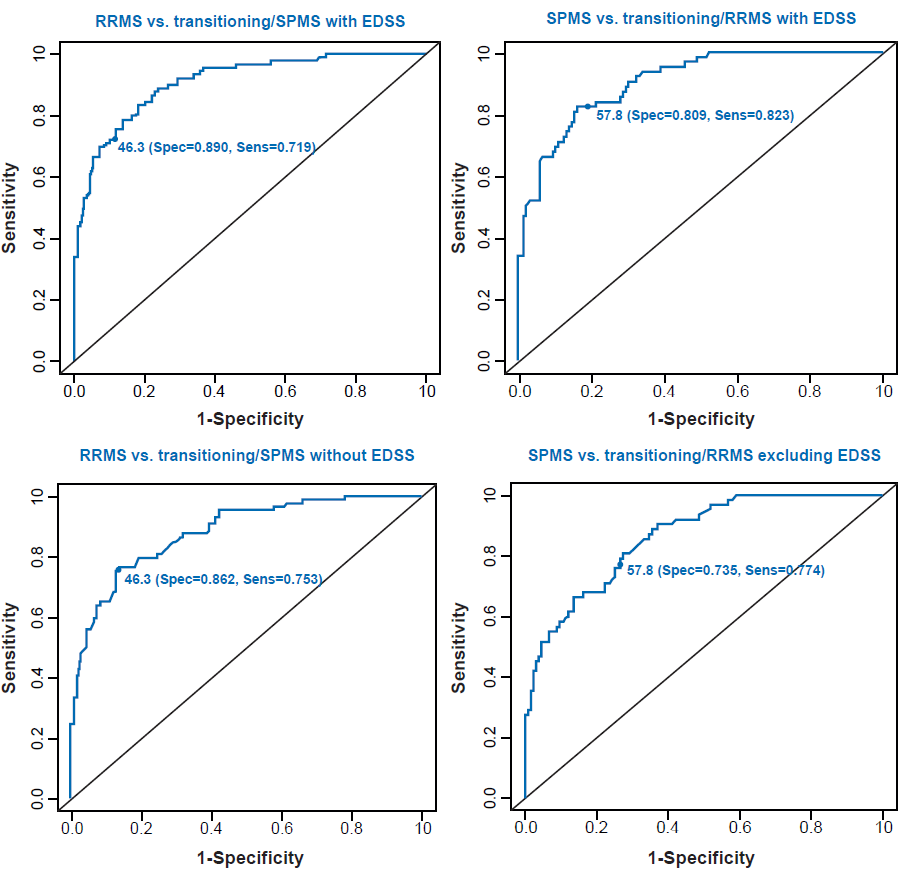


EDSS, Expanded Disability Status Scale; ROC, Receiver Operating Characteristics; RRMS, relapsing–remitting multiple sclerosis; SPMS, secondary progressive multiple sclerosis; Spec, specificity; Sens, sensitivity
