# Supplementary material for: A Physician-Completed Digital Tool for Evaluating Disease Progression (Multiple Sclerosis Progression Discussion Tool): Validation Study
Source: J Med Internet Res. 2020 Feb 12;22(2):e16932. doi: 10.2196/16932 (PMC7055760; doi:10.2196/16932)
Supplement: Multimedia Appendix 9 [file jmir_v22i2e16932_app9.docx]

# Table: Known-groups comparisons for total score

| Known groups | N | Mean (SD) | Median | p-value | Cohen’s *d** |
| --- | --- | --- | --- | --- | --- |
| EDSS | | | | | |
| ≥ 1 and ≤4.5 | 122 | 43.2 (14.0) | 43.4 | <0.001 | 2.173 |
| >4. 5 and < 9.5 | 66 | 70.2 (10.6) | 69.4 |  |  |
| Missing | 10 |  | | | |
| Disease Diagnosis | | | | | |
| RRMS | 89 | 38.1 (12.5) | 34.5 | <0.001 | 2.574 |
| SPMS | 62 | 69.6 (12.0) | 70.0 |  |  |
| Missing | 47 |  | | | |

*Cohen’s *d* effect size

EDSS, Expanded Disability Status Scale; RRMS, relapsing–remitting multiple sclerosis; SD, standard deviation; SPMS, secondary progressive multiple sclerosis
