# Supplementary material for: A Physician-Completed Digital Tool for Evaluating Disease Progression (Multiple Sclerosis Progression Discussion Tool): Validation Study
Source: J Med Internet Res. 2020 Feb 12;22(2):e16932. doi: 10.2196/16932 (PMC7055760; doi:10.2196/16932)
Supplement: Multimedia Appendix 10 [file jmir_v22i2e16932_app10.docx]

# Table: Known-groups comparisons for section scores

| Known groups | N | Section Mean (SD) | | | Median | p-value* | | Cohen’s *d* | |
| --- | --- | --- | --- | --- | --- | --- | --- | --- | --- |
| Disease activity | | | | | | | | | |
| EDSS Score | | | | | | | | | |
| ≥ 1 and ≤4.5 | 122 | 0.674 (0.132) | | 0.750 | | <0.001 | | 0.591 | |
| >4. 5 and <9.5 | 66 | 0.735 (0.060) | | 0.750 | |  |  |  |  |
| Missing | 10 |  | | | | | | | |
| Disease Diagnosis | | | | | | | | | |
| RRMS | 89 | 0.671 (0.134) | | 0.750 | | 0.002 | | 0.550 | |
| SPMS | 62 | 0.730 (0.069) | | 0.750 | |  | |  | |
| Missing | 47 |  | | | | | | | |
| Symptoms | | | | | | | | | |
| EDSS Score | | | | | | | | | |
| ≥ 1 and ≤4.5 | 122 | 0.273 (0.234) | | | 0.286 | <0.001 | | 1.288 |  |
| >4. 5 and < 9.5 | 66 | 0.549 (0.192) | | | 0.578 |  |  |  |  |
| Missing | 10 |  | | | | | | |  |
| Disease Diagnosis | | | | | | | | | |
| RRMS | 89 | 0.206 (0.208) | | | 0.143 | <0.001 | | 1.616 | |
| SPMS | 62 | 0.546 (0.212) | | | 0.596 |  |  |  |  |
| Missing | 47 |  | | | | | | | |
| Impacts | | | | | | | | |  |
| EDSS Score | | | | | | | | |  |
| ≥ 1 and ≤4.5 | 122 | 0.369 (0.226) | | | 0.429 | <0.001 | | 1.383 |  |
| >4. 5 and < 9.5 | 66 | 0.696 (0.246) | | | 0.643 |  |  |  |  |
| Missing | 10 |  | | | | | | |  |
| Disease Diagnosis | | | | | | | | | |
| RRMS | 89 | 0.296 (0.238) | | | 0.286 | <0.001 | | 1.599 | |
| SPMS | 62 | 0.681 (0.243) | | | 0.643 |  |  |  |  |
| Missing | 47 |  | | | | | | | |
| Clinical variables | | | | | | | | | |
| EDSS Score | | | | | | | | | |
| ≥ 1 and ≤4.5 | 122 | 0.408 (0.226) | 0.400 | | | <0.001 | 1.926 | | |
| >4. 5 and < 9.5 | 66 | 0.821 (0.202) | 0.800 | | |  |  |  |  |
| Missing | 10 |  | | | | | | | |
| Disease Diagnosis | | | | | | | | | |
| RRMS | 89 | 0.351 (0.216) | 0.200 | | | <0.001 | 2.232 | | |
| SPMS | 62 | 0.813 (0.198) | 0.800 | | |  |  |  |  |
| Missing | 47 |  | | | | | | | |

EDSS, Expanded Disability Status Scale; RRMS, relapsing–remitting multiple sclerosis; SD, standard deviation; SPMS, secondary progressive multiple sclerosis
