# Supplementary material for: A Physician-Completed Digital Tool for Evaluating Disease Progression (Multiple Sclerosis Progression Discussion Tool): Validation Study
Source: J Med Internet Res. 2020 Feb 12;22(2):e16932. doi: 10.2196/16932 (PMC7055760; doi:10.2196/16932)
Supplement: Multimedia Appendix 11 [file jmir_v22i2e16932_app11.docx]

# Table: Item correlations with total score and item correlations with physician diagnosis

| Item | Correlation with total score [1,2] | Correlation with physician diagnosis [1] |
| --- | --- | --- |
| Ambulatory symptoms | 0.773 | 0.623 |
| Coordination and balance symptoms | 0.757 | 0.574 |
| Motor symptoms | 0.736 | 0.616 |
| Impact on self-care | 0.723 | 0.563 |
| Impact on mobility | 0.713 | 0.597 |
| Impact on hobbies and leisure time | 0.684 | 0.507 |
| Impact on other daily activities | 0.649 | 0.424 |
| Impact on paid and unpaid work (e.g. voluntary work) | 0.584 | 0.493 |
| Cognitive symptoms | 0.569 | 0.431 |
| Sensory symptoms | 0.549 | 0.303 |
| Fatigue | 0.540 | 0.368 |
| Recovery from most recent relapse | 0.482 | 0.535 |
| Bladder and bowel symptoms | 0.473 | 0.367 |
| Pain | 0.379 | 0.244 |
| Number of relapses in the past six months | -0.296 | -0.303 |
| Speech symptoms | 0.275 | 0.152 |
| Visual symptoms | 0.219 | 0.195 |
| Presence of relapses in the past six months | -0.027 | 0.195 |

[1] Spearman correlations.

[2] Sum of items used in the calculation of the section/total score is used instead of the actual score.

*Item-scale correlation corrected for overlap (relevant item removed from its scale for correlation).
